# An enhanced clot growth rate before in vitro fertilization decreases the probability of pregnancy

**DOI:** 10.1371/journal.pone.0216724

**Published:** 2019-05-23

**Authors:** A. N. Balandina, E. M. Koltsova, T. A. Teterina, A. G. Yakovenko, E. U. Simonenko, A. V. Poletaev, I. V. Zorina, A. M. Shibeko, T. A. Vuimo, S. A. Yakovenko, F. I. Ataullakhanov

**Affiliations:** 1 Dmitry Rogachev National Research Center of Pediatric Hematology, Oncology and Immunology, Moscow, Russia; 2 Center for Theoretical Problems of Physicochemical Pharmacology, Moscow, Russia; 3 AltraVita IVF clinic, Moscow, Russia; 4 Lomonosov Moscow State University, Moscow, Russia; 5 Moscow Institute of Physics and Technology, Dolgoprudny, Russia; Zhejiang University School of Medicine Women’s Hospital, CHINA

## Abstract

**Background:**

The shift towards hypercoagulation during in vitro fertilization (IVF) can lead to the impairment of embryo implantation and placental blood circulation, which is believed to be a factor in an unsuccessful IVF cycle.

**Objectives:**

To assess coagulation in women with infertility before the start of an IVF cycle and during treatment to reveal the association between coagulation imbalance and IVF outcome.

**Patients/Methods:**

We conducted a prospective cohort observational study including 125 participants who underwent fresh IVF cycles. Blood samples were collected at five time points: before IVF, one week after the start of controlled ovarian stimulation (COS), on the day of follicular puncture, on the day of embryo transfer (ET) and one week after ET. Coagulation tests (clotting times: activated partial thromboplastin time (APTT) and prothrombin; fibrinogen and D-dimer concentrations; thrombodynamics) were performed.

**Results:**

Women with an elevated clot growth velocity (>32.3 μm/min, detected by thrombodynamics) before IVF demonstrated a higher risk of negative IVF outcomes (adjusted RR = 1.38; 95% CI 1.28–1.49; P<0.001). During the procedure, we observed increases in prothrombin, fibrinogen and D-dimer concentrations, a slight shortening of APTT and a hypercoagulation shift in the thrombodynamics parameters. The hemostasis assay values during COS and after ET had no associations with IVF outcomes.

**Conclusions:**

Hypercoagulation in the thrombodynamics before the start of IVF treatment was associated with negative IVF outcomes. After the start of COS, all tests demonstrated a hypercoagulation trend, but the hypercoagulation did not influence IVF outcome. This research is potentially beneficial for the application of thrombodynamics assay for monitoring hemostasis in infertile women prior to an IVF procedure with the goal of selecting a group requiring hemostasis correction to increase the chances of pregnancy.

## Introduction

Infertility in women is often accompanied by a hypercoagulable state. The rates of thrombophilia [1,2] and circulating microvesicle concentrations [3] are higher and protein C activity is lower [4] in women with conception problems. In vitro fertilization (IVF) can enhance these procoagulant changes in infertile women mostly as a result of high-dose hormonal therapy. The main changes in the coagulation system observed during IVF are increases in coagulation factor concentrations, decreases in coagulation inhibitor concentrations and increases in thrombosis marker (D-dimers and TAT-complex) concentrations [5–10]. Global coagulation assays reveal hypercoagulation during controlled ovarian stimulation (COS) treatment [11,12]. As a result of this hypercoagulation, IVF leads to a 2-3-fold increased risk of venous thromboembolic events (VTEs) [13,14]. Moreover, hypercoagulation induced by IVF can influence IVF outcome; the shift in the coagulation system towards hypercoagulation can lead to the impairment of embryo implantation and of placental blood circulation, which is believed to be one of the causes of infertility and recurrent miscarriage [15–19] and can thus be the cause of an unsuccessful IVF cycle. There is some evidence that low-molecular-weight heparin (LMWH) in prophylactic doses prescribed around the time of embryo implantation increases the chances of conception and a positive pregnancy outcome [20], which also confirms the connection between hemostasis and IVF success. However, the actual mechanism of the heparin effect on IVF outcome is still unclear because, apart from its anticoagulation properties, heparin is believed to improve endometrial receptivity, endometrial stromal cell decidualization, and trophoblast adhesion and invasiveness [21]. Notably, the routine use of LMWH for improvement of conception and live birth rates is not recommended due to an insufficient evidence base. However, treatment strategies still vary between countries and hospitals, and some of them routinely perform IVF cycles with adjuvant anticoagulation.

The causes of IVF failure are various, and it is important to remember that altered coagulation dynamics may be responsible for only a proportion of IVF failures. The strategy of administering heparin during IVF according to a patient’s previous IVF or pregnancy outcome and complications (such as a history of IVF failures, miscarriages, or preeclampsia) does not take into account the causative disease mechanism and thus limits the ability to determine the effect of this treatment [22]. Finding a way to distinguish a group that might actually benefit from anticoagulants and establishing the individual treatment strategies stratified by mechanism rather than by outcome for such patients is a goal that needs to be achieved.

Although all women planning IVF undergo routine hemostasis testing, in practice, the reproductive specialist rarely takes the test results into account. There are two explanations for this. First, the importance of deviations in the hemostasis system for the subsequent success of the IVF procedure is not recognized by many practicing physicians. Second, routine coagulation tests are able to show only hypocoagulation derived from serious deficiencies in clotting factors, while hypercoagulation, which has a major impact on conception, remains unnoticed. Global coagulation assays are considered to be most suitable for both the detection of hypercoagulation and monitoring of anticoagulant prophylaxis or therapy [23,24].

It is well known that global hemostasis assays are informative for the assessment of hypercoagulation in pregnancy [25]. A new global coagulation assessment, namely, thrombodynamics, seems to be suitable for both the detection of hypercoagulation and monitoring of anticoagulant prophylaxis or therapy [26–29], including the monitoring of anticoagulant prophylaxis in pregnancy [30].

The objective of this study was to assess the coagulation state with standard coagulation assays (APTT, prothrombin, fibrinogen and D-dimer concentration) and a global coagulation assay (thrombodynamics) in women with infertility before the start of an IVF cycle and during treatment to reveal the association between coagulation imbalance and IVF outcome.

## Materials and methods

### Study design and study population

Women undergoing IVF treatment in the AltraVita IVF clinic (Moscow, Russia) were included in this prospective noninterventional cohort study between October 2015 and February 2018. All participants met the following inclusion criteria: 1) age over 20 years and less than 40 years; 2) a plan to undergo an IVF protocol with fresh embryo transfer (ET); 3) the absence of any trauma or surgical treatment up to 90 days before the start of treatment; 4) the absence of malignancy or autoimmune diseases currently or previously; 5) the absence of polycystic ovarian syndrome; 6) the absence of ongoing anticoagulation treatment at the start of IVF treatment; and 7) a plan for anticoagulant prophylaxis to be prescribed after oocyte retrieval or ET. The exclusion criteria were as follows: 1) the appearance of contraindications for the start of IVF treatment; 2) a decision to proceed with the IVF protocol with frozen ET; 3) an interruption in the IVF protocol due to ovarian hyperstimulation syndrome (OHSS); and 5) a decision of the physician to proceed without anticoagulation treatment after ET.

Oocytes were obtained by transvaginal follicular puncture (FP) under ultrasound guidance 36 hours after ovulatory human chorionic gonadotropin (hCG) administration. Intracytoplasmic sperm injection (ICSI) or intracytoplasmic morphologically selected sperm injection (IMSI) procedures were implemented for the fertilization of the obtained oocytes. For ET, the blastocysts of AA, AB, BA and BB quality according to Gardner DK were used [31]. The clinical outcome of the investigation was the success or failure of the IVF protocol, evaluated by ultrasound investigation. Clinical pregnancy was confirmed when an intrauterine gestational sac was revealed in a transvaginal ultrasound scan 5 weeks after ET [32]. Here and below, a confirmed pregnancy is referred to as a “positive IVF outcome”, and its absence is referred to as a “negative IVF outcome”.

We recruited a group of 25 healthy women (aged 20–39 years) with term and healthy children as a result of uncomplicated pregnancy to serve as the control group for thrombodynamics. The inclusion criteria were as follows: 1) no history of miscarriages; 2) no preeclampsia during any of pregnancy; 3) a child/children who were not conceived by artificial reproductive technologies; 4) a time period since the last birth of no less than 12 months; 5) the absence of a thrombotic or hemorrhagic history, as well as diagnosed cardiovascular diseases; 6) no respiratory infections for at least the last two weeks before inclusion; 7) no current therapy with anticoagulants or antiplatelet agents; and 8) no current hormonal treatment (including oral contraceptives).

### Treatments

COS was initiated on day 2 or 3 of a spontaneous or stimulated cycle. An initial dose of 150–300 IU recombinant follicle-stimulating hormone (FSH) and/or highly purified human menopausal gonadotropin (hMG) was administered. From day 6 onward, the gonadotropin dose was estimated according to serum estradiol (E2) levels and a transvaginal ultrasound scan. When the leading follicle reached 13–14 mm, a gonadotropin-releasing hormone (GnRH) antagonist was administered at 0.25 mg/d. Final oocyte maturation was triggered with 5,000–10,000 IU highly purified hCG or recombinant hCG as soon as the mean diameters of two follicles reached 18 mm.

Administration of micronized progesterone (P; 600 mg/d, vaginally) was initiated on the day of FP. After ET, some patients received oral estradiol valerate (EV; 2–6 mg/d). If pregnancy was achieved, administration of P and EV was maintained until gestational week 12.

LMWH (enoxaparin sodium, 40 mg/d, equivalent to 4000 IU of anti-Xa activity or Nadroparin calcium 0.3 ml/d, equivalent to 2850 IU of anti-Xa activity) was started one day after FP or one day after ET. If pregnancy was achieved, administration of LMWH was maintained until gestational week 2. LMWH in prophylactic doses is given routinely during IVF in most reproduction centers in Russia. The timing of the prescription (after oocyte retrieval or after ET) is not stated clearly in any guidelines, so the treatment start time completely depends on the clinician’s decision.

### Blood sampling and preparation

Blood samples for monitoring coagulation were collected at five time points: before IVF (Point 1, P1) on the second menstrual cycle day; one week after the start of COS (Point 2, P2); on the day of FP (Point 3, P3); on the day of ET (Point 4, P4); and one week after ET (Point 5, P5). Blood in P5 was sampled 2–9 hours after LMWH injection. Blood from the healthy women included in the control group for thrombodynamics was obtained on a random menstrual cycle day. Blood was drawn into 4.5 ml vacuum plastic tubes (Vacuette, Greiner Bio-One, Kremsmunster, Austria) with 3.2% sodium citrate buffer at a 9:1 blood:anticoagulant volume ratio. Whole blood was processed by centrifugation at 1,600 g for 15 minutes. A portion of the plasma was frozen in liquid nitrogen and then stored at -80°C for further APTT, prothrombin, fibrinogen and D-dimer assays. The remaining plasma was repeatedly processed by centrifugation at 10,000 g for 5 minutes and used for the thrombodynamics assays.

### Coagulation assays

APTT, TT, prothrombin, fibrinogen and D-dimer assays were performed with automatic coagulometer ACL TOP 700 and HemosIL reagents (Instrumentation Laboratory, USA, MA).

A thrombodynamics assay was performed with a thrombodynamics analyzer and thrombodynamics kit (HemaCore LLC, Russia) [26]. This method is based on the photoregistering of spatial fibrin clot growth after activation of clotting with the immobilized tissue factor-bearing surface. Briefly, 120 μl of plasma was added to a micro tube with dried CTI (0.2 mg/mL) and HEPES (30 mM, pH 7.2–7.4). Afterward, the sample was incubated for 3 minutes at 37°C in the special hole of the thermostat of the analyzer. Subsequently, plasma was supplemented with dried calcium acetate (20 mM), and the sample was immediately placed in a plastic cuvette. Clotting was activated using a plastic plate that was covered with covalently attached TF (100 pmole/m^2^) [33].

**Fig 1:**
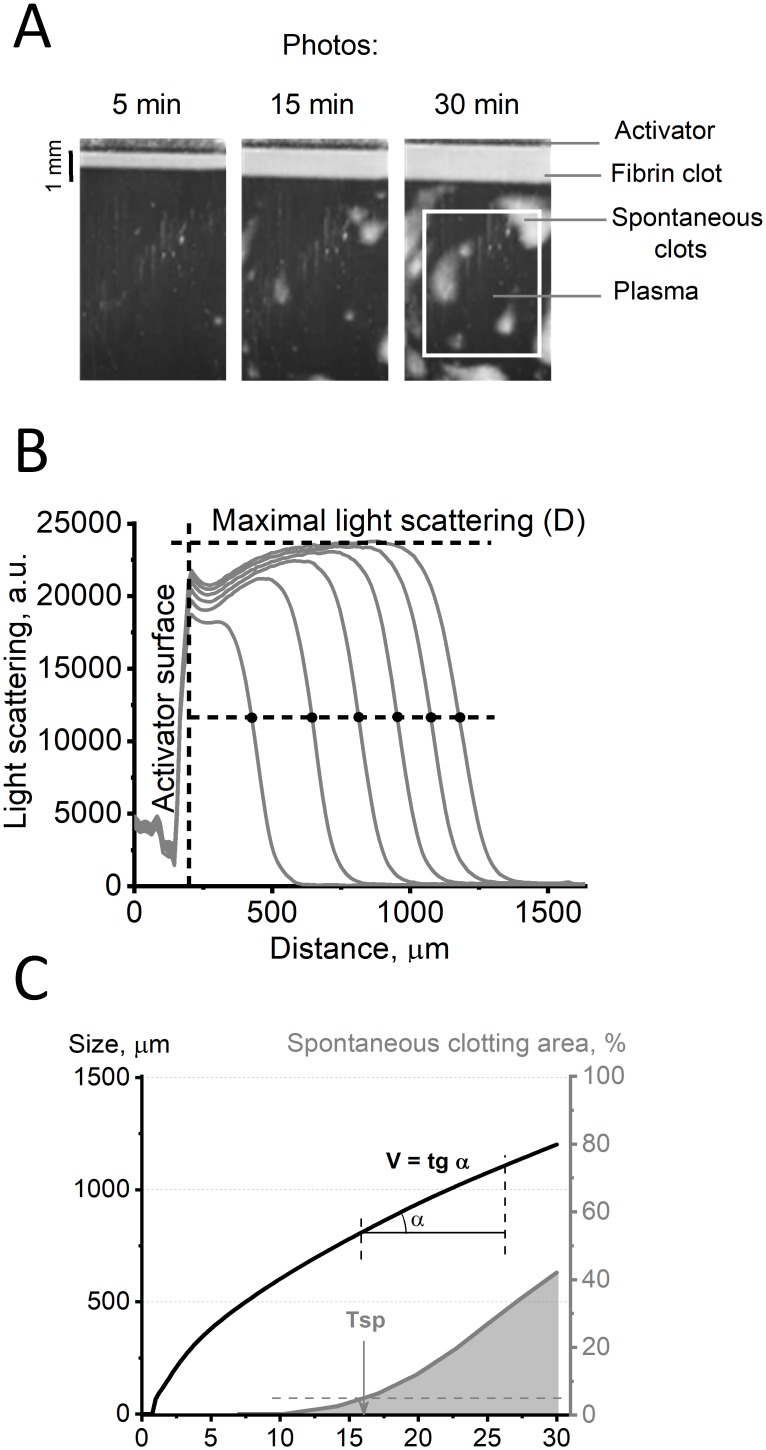
The thrombodynamics assay principle. (A) Photographs of fibrin clot growth. The edge of the activator on the top of the pictures is covered with immobilized tissue factor. The clot starts to grow from the edge of the activator to the bulk of the plasma. The process of fibrin clot formation is recorded in time-lapse video microscopy mode by means of a dark-field light scattering method. The obtained series of photos shows how the form, size, and density of the fibrin clot changes over time. The white frame indicates the spontaneous clotting calculation region. (B) Light scattering profiles of fibrin clots. The coordinates of the fibrin front are the coordinates of the half-maximal light scattering intensity. (C) Plot of clot growth versus time, representing the parameters V and Tsp of thrombodynamics.

Experiments were performed using a video microscopy system that allows the observation of the spatial dynamics of fibrin clot growth. Fibrin clot formation was detected by red light scattering. Scattered light was detected by a digital camera every 6 seconds for 30 minutes (Fig 1A). The light scattering is linearly related to the fibrin concentration [34], so we presented these data as the fibrin concentration in arbitrary units. For each light scattering profile (Fig 1B), the clot size was determined as the coordinate of the half-maximal light scattering intensity (Fig 1C). The average area of spontaneous clots as a function of time (Fig 1C) was calculated within the measurement region indicated in Fig 1A (white frame).

The clot size as a function of time was used to calculate the following parameters: the velocity of clot growth (V), namely, the mean clot growth velocity over a 10-minute interval of the stationary growth period or before spontaneous clotting (the formation of clots in the space of the experimental cuvette not associated with the main clot growth). With this curve, the spontaneous clotting time (Tsp) was calculated, namely, the time to filling 5% of the analyzed cuvette area by spontaneous clots (practically, we used the reversed parameter 1/Tsp; it was equal to zero with no spontaneous clotting and increased if spontaneous clotting occurred earlier). Parameter D was calculated as the maximal light scattering of the clot.

The thrombodynamics assay reproducibility (the total coefficients of variation for all the parameters) was measured as previously reported [35] and was 10% for V. Spontaneous clots were absent in healthy volunteers, so the variation coefficient for Tsp was obtained by mimicking hypercoagulation by the addition of fXIa to plasma [36] and was 14%.

### Reference values for coagulation assays

The reference ranges for APTT, prothrombin, fibrinogen and D-dimer assays were obtained from the clinical hemostasis laboratory of the National Research Center of Pediatric Hematology, Oncology and Immunology and are the manufacturer’s reference ranges that were approved in-house.

Reference ranges for thrombodynamics parameters were calculated as the 5–95% interval of the control group values.

### Potential sources of bias

The main potential sources of bias were the heterogeneity of the patients’ anamnesis, treatment during the IVF cycle and variability in transferred embryo quality. To address these potential sources of bias, we compared the groups with positive and negative IVF outcomes according to their clinical characteristics prior to the main analysis. Additionally, we had a significant loss of follow-up during the longitudinal phase of the study. To address this potential source of bias, we included only the patients who had a complete set of timepoints into the analysis and performed the comparison with pairwise statistical instruments.

### Statistical analysis

The number of cases in the area during the study period determined the sample size.

For the purposes of comparison of baseline characteristics, COS parameters and IVF data between pregnant and nonpregnant groups, we used descriptive statistical methods (the median and interquartile range (IQR, 25–75 percentile values)) and the Mann-Whitney U test or Fisher’s exact test for categorical variables. The same analysis was performed for hemostasis assay parameters. To obtain the optimal cut-off levels for diagnostic tests, a ROC analysis of the data exceeding the reference range, calculated with the control croup, was performed. The test value with the maximum Youden index was chosen as the cut-off. After dichotomization of the data, relative risk (RR) values were calculated. Potential cofounders were tested by the stratification method, and RRs were subsequently adjusted using the Cochran-Mantel-Haenszel method [37]. Due to the nonnormal distribution of the data, the difference between timepoints was evaluated using Friedman ANOVA for repeated measurements and the pairwise Wilcoxon signed rank test. For comparisons of categorical variables, we used Fisher’s exact test. A P value <0.05 was considered to be significant.

To reveal any interassay correlations, we performed Spearman correlation analysis. If R_Spearman_ was greater than 0.6 (P<0.05), we considered the correlation significant.

Statistical analysis was performed using Origin Pro 8 (OriginLab Corp., USA) Software. ROC analysis, risk ratio calculation and adjustment, and Friedman ANOVA were performed with MedCalc Statistical Software v. 14.8.1 (MedCalc Software, Ostend, Belgium).

### Ethical approval

The study was approved by the Local Ethics Committee of the AltraVita IVF clinic (LLC “IVF Center”)(Protocol #2). The Ethics Committee reviewed all forms for patients and researchers, as well as documentation for the laboratory instruments used. The informed consent form was considered informative enough for the patient to make an informed decision to participate in the study. The benefits of the study were found to exceed the potential risks for the patient. After the protocol start, written informed consent was obtained from all participants.

## Results

### Hemostasis before IVF

**Fig 2:**
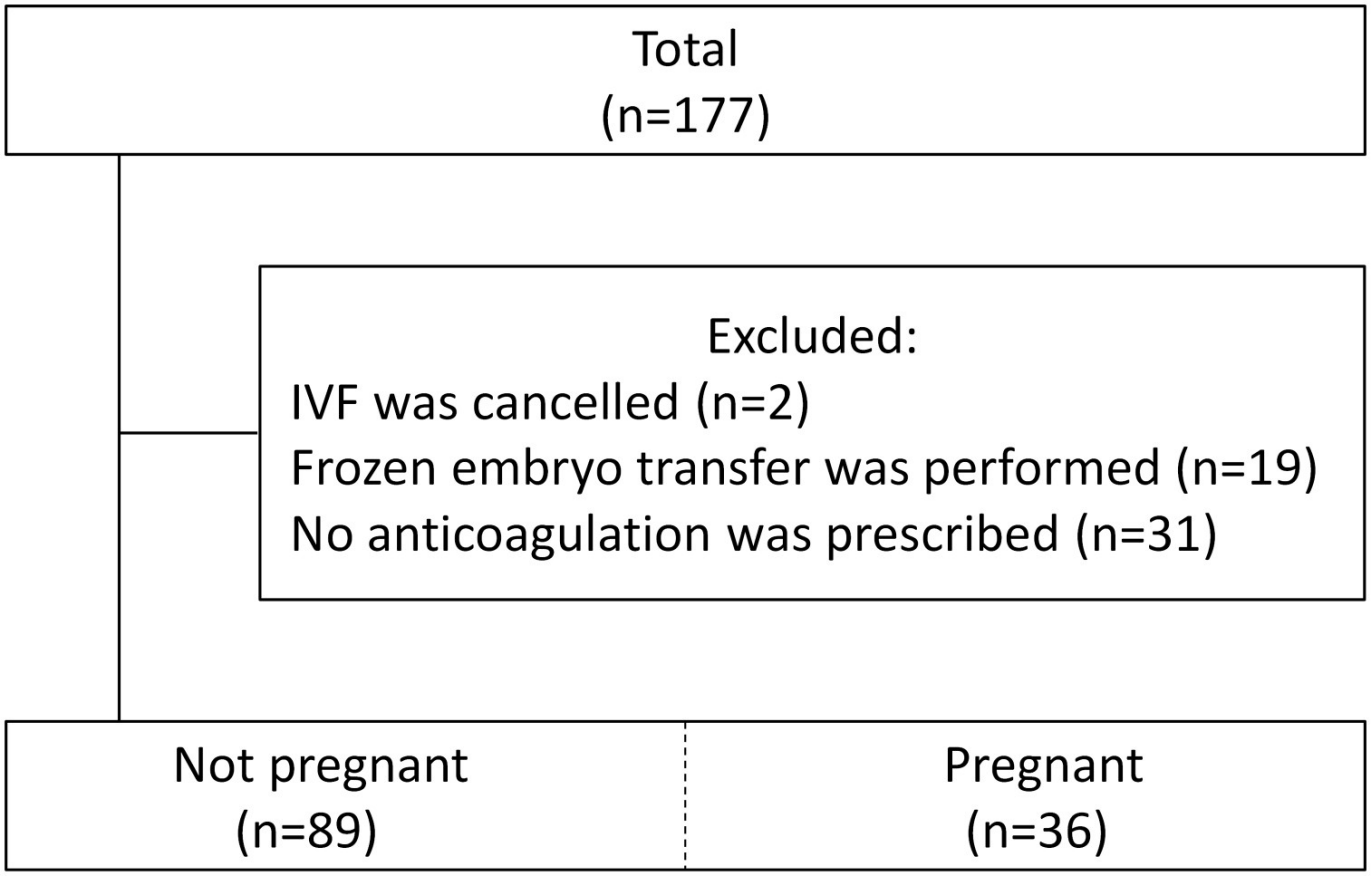
Flow chart depicting the patients’ recruitment and IVF outcomes. P1 = Point 1 (before the start of IVF).

Of the 177 women, 52 were excluded during the analysis: 2 patients had not started an IVF protocol, 19 patients had undergone frozen ET (including the patients who developed the OHSS), and 31 patients had decided to proceed without anticoagulation (Fig 2).

The baseline characteristics of the 125 women are displayed in Table 1. Pregnancy was achieved as a result of IVF in 36 women and was not achieved in 89 women. Neither the mean age nor the percent of women aged over 35 y differed significantly between these groups. Body mass index, infertility duration and infertility type distribution (primary or secondary) did not differ between the positive and negative IVF outcome groups, nor did infertility causes. One patient with arterial hypertension (with a maximum arterial blood pressure of 160/100 mm Hg) was included in the study, and none of the participants had diabetes mellitus; thus, we did not estimate the influence of these factors on pregnancy rates. The percentage of women with clinically compensated hypothyroidism did not significantly differ between groups. Furthermore, the positive and negative IVF outcome groups were not different in terms of hormonal therapy, the oocyte mean number or the obtained blastocyst mean number. Some of the patients had two embryos transferred (7 women in the IVF-negative group and 6 women in the IVF-positive group), but the median number of embryos transferred did not differ significantly between the groups.

**Table 1:** Baseline characteristics, COS parameters, IVF data.

Characteristic	Non-pregnant	Pregnant	P value†
*Baseline characteristics*			
Patients (n)	89	36	NA
Age (y)	32 (29–36)	32 (29–35)	0.326
Advanced reproductive age (>35 y) (n/%)	24/27%	6/16%	NS^#^
BMI (kg/m^2^)	22.7 (20.5–26.0)	22.3 (21.0–25.8)	0.860
Duration of infertility (y)	5 (4–8)	4.5 (3.5–7.5)	0.149
Secondary infertility (n/%)	37/42%	13/36%	NS^#^
*Cause of infertility* ^a^			
Male factor (n/%)	50/56%	20/56%	NS^#^
Tubal factor (n/%)	55/62%	26/72%	NS^#^
Endometriosis/chronic endometritis (n/%)	39/44%	20/56%	NS^#^
Endocrine factor ^b^ (n/%)	33/37%	14/36%	NS^#^
Number of previous IVF cycles	0 (0–1)	0 (0–1)	NS
Number of patients having no previous IVF	60/67%	24/66%	NS^#^
*Pre-existing medical conditions*			
Hypertension (n/%)	0/0%	1/2.7%	-
Diabetes (n/%)	0/0%	0/0%	-
Compensated hypothyroidism ^c^ (n/%)	20/22%	3/8%	NS^#^
Thromboses in anamnesis (n/%)	0/0%	0/0%	-
*Controlled ovarian stimulation (COS)*			
Length of stimulation (d)	9 (8–9)	8 (8–9)	0.794
Total FSH dose (IU)	2,025 (1,750–2,250)	2,025 (1,650–2,250)	0.852
Total hCG dose (IU)	9,000 (6,500–10,000)	9,000 (6,500–10,000)	0.400
*IVF*			
No. of retrieved oocytes (n)	11 (8–17)	10 (7–16)	0.666
No. of 2PN (two pro-nucleate) embryos (n)	8 (5–11)	8 (5–12)	0.766
No. of blastocysts (n)	4 (1–6)	4 (2–6)	0.628
No. of transferred embryos (n)	1 (1–1)	1 (1–1)	0.147

Continuous data: median (interquartile range); other values are presented as the number (percentage).

^†^P value represents the difference between nonpregnant and pregnant women (Mann-Whitney U test or Fisher’s criterion^#^). NA = not applicable.

^a^ Many participants had more than one diagnosis.

^b^ Includes ovulatory disorders defined per the World Health Organization Classification of Ovulatory Disorders, group I or II criteria (i.e., hypothalamic–pituitary–gonadal axis failure or dysfunction) and decreased ovarian reserve defined as any of the following: day-3 FSH (follicle-stimulating hormone)>10, AMH (anti-Mullerian hormone)≤8 pM/L, total antral follicle count <6 or prior poor response (defined as prior IVF cycle cancelation due to poor Q8 response or fewer than four oocytes retrieved).

^c^ Normalization of TSH (thyroid stimulating hormone) before IVF.

The parameters of the blood cell count and hormones were within the normal range for all of the patients and did not differ significantly between the positive and negative IVF outcome groups, except for a slight difference in the platelet count (S1 Table).

**Fig 3:**
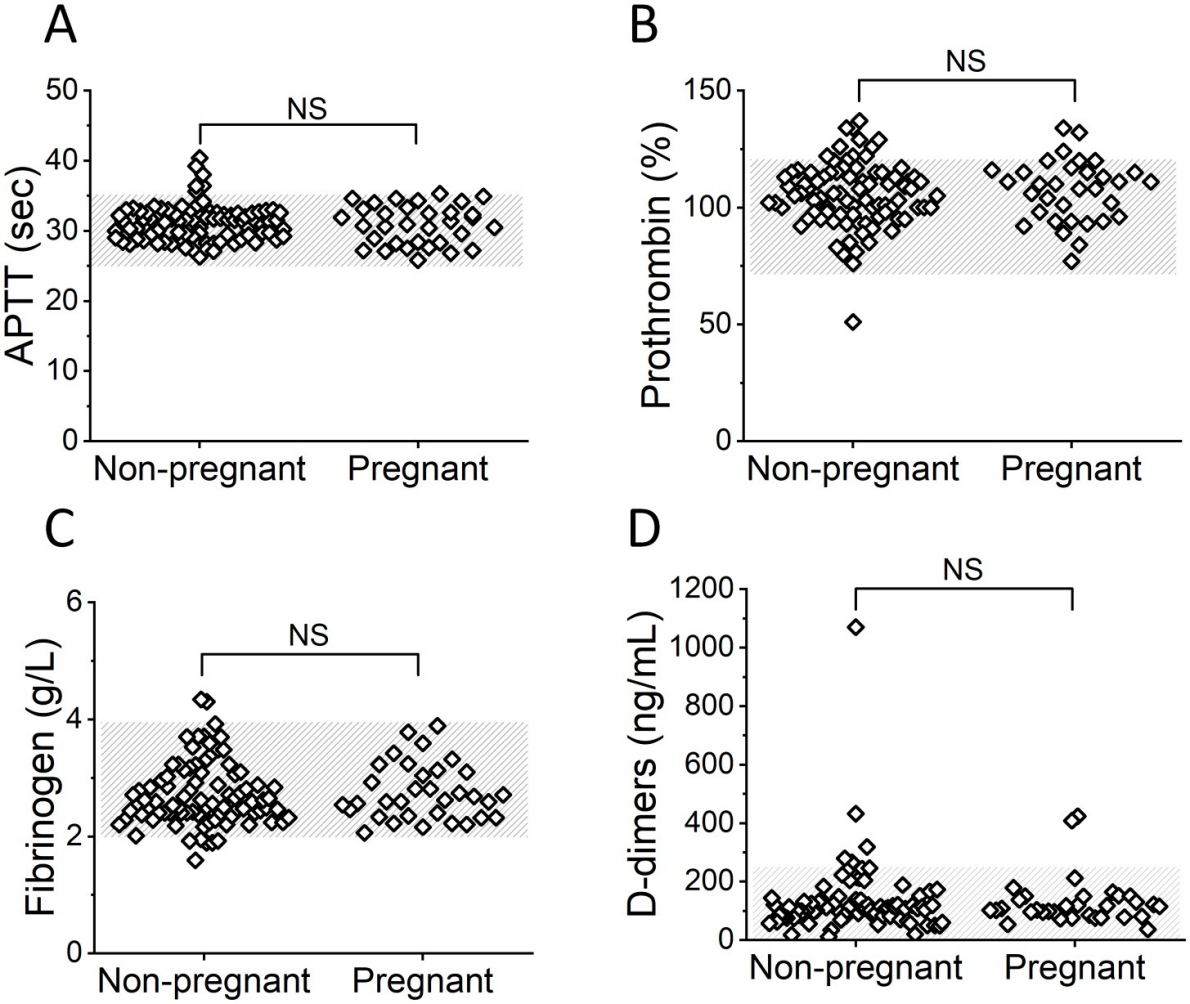
Standard coagulation test results of patients before IVF. (A) APTT, (B) prothrombin, (C) fibrinogen, (D) D-dimers. The shaded area indicates the normal range. NS indicates a nonsignificant difference; Mann-Whitney U test) between nonpregnant (n = 89) and pregnant (n = 36) women.

In general, the hemostasis parameters did not differ significantly between the nonpregnant and pregnant groups (Fig 3 and S2 Table).

**Fig 4:**
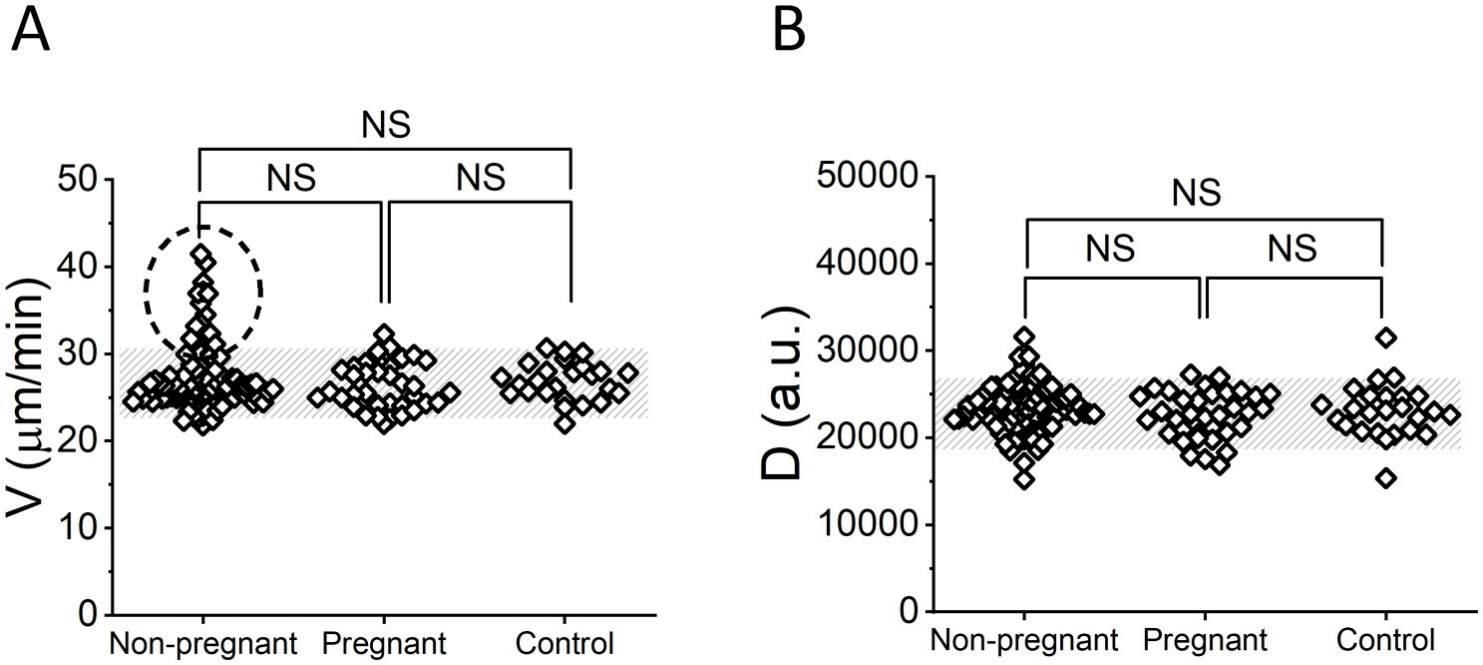
Thrombodynamics results of patients before IVF. (A) V, (b) Maximal light scattering (D). The shaded area indicates the normal range, calculated as a 5–95% interval for the control group (n = 25). NS indicates nonsignificant difference; Mann-Whitney U test). The dashed circle represents patients with hypercoagulation based on the high clot growth velocity V in the thrombodynamics compared with the reference range.

Parameters V and D in the thrombodynamics did not differ between the nonpregnant and pregnant groups or between the IVF and control groups (Fig 4 and S2 Table). Neither of the patients had spontaneous clotting. However, we observed a hypercoagulant “tail” for thrombodynamics parameter V in the IVF-negative group (dashed circle in Fig 4).

ROC analysis revealed that for parameter V in the thrombodynamics, the optimal cut-off value was 32.3 μm/min (S3 Table). Risk ratio analysis demonstrated that the crude relative risk of a negative IVF outcome for women with hypercoagulation (V>32.3 μm/min) was 1.4 times higher than that for women with V≤32.3 μm/min (RR_crude_ = 1.41; 95% CI 1.20–1.67; P<0.001) (Table 2). We adjusted the crude RR for potential clinical confounders, namely, age, BMI, type of infertility, number of previous IVF cycles and number of retrieved oocytes. These confounders were included because they could potentially influence both the IVF outcome and hemostasis. We categorized age and BMI according to the following criteria: advanced reproductive age (age≥35 y), obesity (BMI≥25) and the boundary level of retrieved oocytes according to Magnusson et al. [38] for which the risks of OHSS are considered to be significantly elevated (N>18). The adjusted RR for women with hypercoagulation before the start of IVF was only slightly decreased, as there were no significant effects of the confounders (RR_adj_ = 1.38; CI 1.28–1.49; P<0.001).

**Table 2:** Crude or adjusted relative risk (RR) for the group with hypercoagulation (V>32.3 μm/min) at P1 according to thrombodynamics analysis.

	N	RR	95% CI	P
**Crude RR**	125	1.41	1.20–1.67	<0.001
*Age*				
≥35	41	1.24	0.88–1.77	
<35	84	1.44	1.15–1.81	
**Adjusted RR**		1.39	1.22–1.58	<0.001
*BMI*				
≥25	35	1.41	0.95–2.08	
<25	88	1.35	1.09–1.68	
**Adjusted RR**		1.39	1.23–1.58	<0.001
*Infertility type*				
Primary	75	1.40	1.07–1.82	
Secondary	50	1.33	1.00–1.76	
**Adjusted RR**		1.39	1.22–1.58	<0.001
*Number of previous IVF*				
0	84	1.35	1.05–1.73	
>0	41	1.42	1.04–1.95	
**Adjusted RR**		1.40	1.23–1.59	<0.001
*Number of retrieved oocytes*				
>18	25	1.37	0.90–2.08	
≤18	100	1.38	1.12–1.71	
**Adjusted RR**		1.40	1.23–1.59	<0.001
**Overall adjusted RR**		1.38	1.28–1.49	<0.001

Weighted pooled relative risk per the fixed effects model (adjusted RR) was calculated using the Cochran-Mantel-Haenszel test (based on Mantel & Haenszel, 1959)

We analyzed the individual evolution of the V parameter from P1 to P5 (S1 Fig) but failed to find any clear patterns. If a patient started her treatment in a state of hypercoagulation, her coagulation could either stay this state or normalize. We observed the same tendency in the patients who started treatment with normal coagulation, namely, they could stay within the normal range or shift towards hypercoagulation.

### Effect of controlled ovarian stimulation on hemostasis

The patients’ numbers in P2, P3, P4 and P5 were 108, 88, 94 and 68, respectively. The average follow-up time for each patient was 22 days. For the purposes of statistical clarity for each test parameter, we limited the sample to the patients who were tested at all five time points. Friedman ANOVA for repeated measurements showed that for all investigated parameters, we could reject the idea that all of the differences between the timepoints were due to random sampling and conclude instead that at least one of the timepoints differed from the rest. Furthermore, we performed a pairwise Wilcoxon signed rank test to reveal the significance of the differences between sequential timepoints.

**Fig 5:**
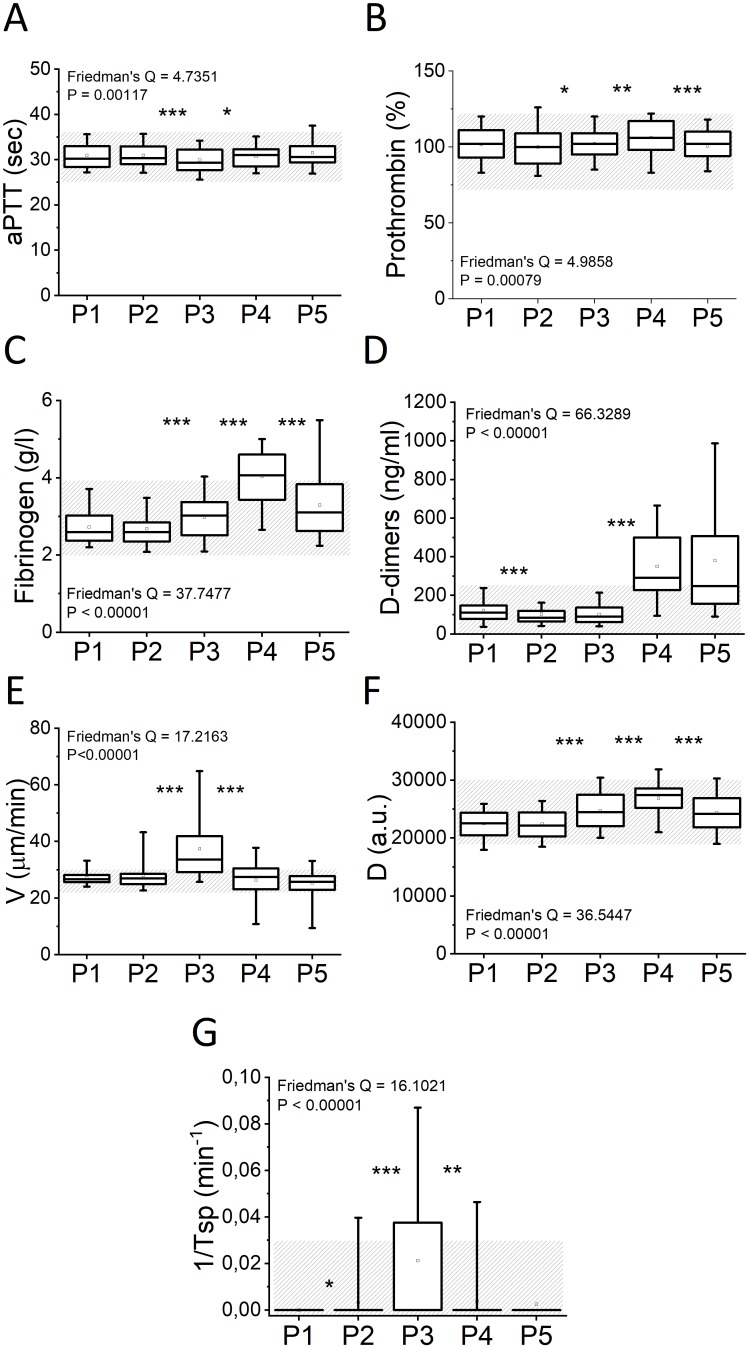
Coagulation test dynamics of patients during IVF. (A) APTT (N = 47), (B) prothrombin (N = 45), (C) fibrinogen (N = 45), (D) D-dimers (N = 44), (E) V in thrombodynamics (N = 53), (F) D in thrombodynamics (N = 50), and (G) 1/Tsp in thrombodynamics (N = 54): before IVF (P1), one week after the start of COS (P2), before FP (P3), before ET (P4) and one week after ET (P5). The shaded area indicates the normal range. The box plots indicate the following parameters: the mean value (the dot inside the box), the 5th and 95th percentiles (the ends of the whiskers), the 25th and 75th percentiles (the bottom and top of the box, respectively), and the median (the horizontal line inside the box). *, ** and *** indicate significant differences (P<0.05, P<0.01 and P<0.001, respectively; pairwise Wilcoxon test) between sequential timepoints.

The dynamics of the coagulation parameters during the IVF cycle (for all of the participants regardless of the IVF treatment outcome) are presented in Fig 5. APTT shortened significantly at P3; however, all values remained within the normal range at all time points. Prothrombin increased significantly throughout COS and was maximal on the day of ET (P4) with a decrease at P5. Most of the values remained within the normal range. The fibrinogen concentration had a similar trend: it increased at P3 and P4 and decreased at P5. The percentage of women with elevated fibrinogen at P4 was 55%. D-dimer dynamics were different from the parameters described above; we observed a slight decrease in D-dimer levels at P2 with a subsequent progressive increase up to P4 and P5 (at P5, the percentage of women with elevated D-dimers was 61%). The decrease in the D-dimer level in P2 is associated with a change in the hormonal phase and is consistent with previous studies [39–41]. The parameters of thrombodynamics demonstrated the progression of hypercoagulation starting at P2, reaching a maximum at P3 (clot growth velocity was elevated in 60% of patients and spontaneous clotting was observed in 38% of patients) and subsequently normalizing at P4 and P5.

We analyzed the differences between hemostasis parameters during IVF treatment (P2-P5) in the positive and negative IVF outcome groups (Table 3). For this calculation, we used the full sample size at each timepoint (N_P2_ = 108; N_P3_ = 88; N_P4_ = 94; N_P5_ = 68). No differences in the parameters of standard tests were revealed between groups at any of the timepoints. Clot growth velocity in the thrombodynamics analysis was elevated in the positive IVF group (at P5, the median clot growth velocity was 27.5 μm/min (IQR 20.1–29.0) in women who achieved pregnancy vs 25.6 μm/min (IQR 23.1–27.2) in women who did not achieve pregnancy, P = 0.049).

**Table 3:** Hemostasis assay parameters during IVF.

Parameter	IVF-positive / IVF-negative P value			
	P2	P3	P4	P5
Standard assays				
APTT (sec)	30.1 (29.1–32.3)/30.7 (29.4–32.5)	29 (27.7–31.7)/30.2 (27.8–32.1)	30.3 (28.5–32)/30.3 (28.8–32.7)	30.4 (28.6–32.1)/31.3 (29.4–33.0)
	P = 0.668	P = 0.295	P = 0.719	P = 0.255
Prothrombin %)	100 (89–113)/103 (94–111)	104 (94–110)/105.5 (99–113)	108 (98–118)/111 (103–122)	99 (90–113)/106 (98–113)
	P = 0.691	P = 0.364	P = 0.284	P = 0.114
Fibrinogen (g/L)	2.74 (2.28–3.02)/2.70 (2.33–3.10)	2.85 (2.41–3.32)/3.01 (2.62–3.38)	3.81 (3.23–4.14)/4.06 (3.28–4.60)	3.05 (2.65–3.80)/3.15 (2.59–3.78)
	P = 0.706	P = 0.276	P = 0.277	P = 0.900
D-dimers (ng/L)	81 (65–103)/94 (68–139)	98 (75–131)/91 (69–139)	288 (213–441)/300 (311–498)	297 (192–542)/259 (156–470)
	P = 0.346	P = 0.613	P = 0.678	P = 0.356
Thrombodynamics				
V (μm/min)	27.6 (25.4–29.2)/27.2 (25.4–28.5)	34.7 (29.47–46.2)/32.1 (28.3–42.9)	28.3 (25.6–35.0)/26.5 (23.4–29.7)	27.5 (20.1–29.0)/25.6 (23.1–27.2)
	P = 0.282	P = 0.557	P = 0.088	P = **0.049**
SpCl (%)	16%/9%	46%/32%	15%/12%	10%/2%
	NS^#^	NS^#^	NS^#^	NS^#^

Test results before IVF (P1), one week after the start of COS (P2), after FP (P3), after ET (P4) and one week after ET (P5) are presented. Continuous data: median (interquartile range); only SpCl is presented as a number (percentage). P values represent differences between nonpregnant and pregnant women (Mann-Whitney U test or Fisher’s criterion^#^). NS = not significant.

The only interassay correlation we observed was the correlation between the D parameter in the thrombodynamics analysis and fibrinogen concentration (R_Spearman_ = 0.77; P<0.001).

## Discussion

Our research was dedicated to assessing the coagulation state of women with infertility diagnoses before the start of and during IVF treatment and to determining whether IVF outcomes are associated with any laboratory coagulation assay result. We found that hypercoagulation with an elevated clot growth velocity in the thrombodynamics analysis before the start of the IVF cycle is associated with an increased risk of a negative outcome of the IVF program. Thus, this test has high potential for application towards identifying a subpopulation of IVF-eligible women who present baseline hypercoagulability and are at high risk of IVF failure.

Our data are consistent with a previous study by Gerotziafas et al., who showed that the procoagulant phospholipid-dependent clotting time (Procoag-PPL) and tissue factor activity (TFa) in plasma before IVF treatment indicated a hypercoagulant shift in the samples of women with unsuccessful outcomes compared with the women who became pregnant [42]. However, in their study, the results of the thrombin generation assay and TEG before the start of IVF were not associated with IVF outcomes [42]. In another paper by El Masry and colleagues [4], it was shown that women who had a negative outcome have a more pronounced decrease in protein C activity than women who became pregnant, which also points to the connection between the initially present procoagulant disorder and a negative IVF outcome.

In our study, the standard coagulation assay values were not associated with IVF outcome before the start of treatment. The D-dimer concentration was elevated in some of the women before IVF treatment, but this elevation did not correlate with the outcome of the cycle. Moreover, in patients who had elevated levels of D-dimers before the start of treatment, these levels remained persistently high during the whole IVF cycle (S2 Fig), which indicates the individual profile of coagulation and fibrinolysis system in such patients, rather than the pathological process. Our finding somewhat contradicts the previously published data about elevated D-dimer levels at the point before r-hCG treatment in women who become pregnant [43]. We found that the D-dimer level at P2 was higher in the nonpregnant group, but the difference was nonsignificant. This may be because the D-dimer levels in general were lower in our study than in the study by Di Nisio et al., which may be due to the different hormone treatment protocols that were used. Other tests (APTT, prothrombin, and fibrinogen) before the start of therapy stayed within the normal range and did not correlate with IVF outcome. The same results for D-dimers and fibrinogen were achieved in a previous study [42]. The difference in platelet count before the start of IVF treatment between the pregnant and nonpregnant groups is an interesting phenomenon. Its association with a low chance of pregnancy achievement seems paradoxical because the cut-off value is within the normal range for the platelet count (230x10^9^/L) (S4 Table). We have not found any information in the literature regarding the adverse effects of high platelet levels, although some of the studies reveal the positive effects of antiplatelet drugs (low-dose aspirin therapy) on the outcome of IVF treatment [44]. However, the system analysis does not prove that these effects are statistically significant [45]. Neither the D-dimer concentration nor platelet count were associated with hypercoagulation in the thrombodynamics analysis at P1 (S3 Fig). Despite the fact that hypercoagulation according to V in the thrombodynamics analysis led to an increased IVF failure risk, we found no clear patterns after the start of COS therapy (S1 Fig). It is possible that changes in the phase of the menstrual cycle (which, in general, leads to hypocoagulation or normalization [39–41] in the results of laboratory tests) or the start of hormonal therapy (which leads to hypercoagulation) can result in individual coagulation patterns in each treated woman. For example, the coagulation pattern can normalize after initial hypercoagulation as a result of a change in the menstrual phase or shift towards hypercoagulation after an initially normal coagulation state as a result of an individual reaction to COS therapy. On average, the hemostasis of patients at P2 remains the same as that before the start of therapy, and only the high-dose trigger causes a significant shift towards hypercoagulation in most patients.

The shift towards hypercoagulation following hormonal therapy has remained consistent with that shown in previous studies [12,42,46,47]. However, the timing of maximum hypercoagulation differs among the assays (the clot growth velocity is maximal on the day of oocyte retrieval; prothrombin and fibrinogen levels are maximal on the day of ET, and D-dimer levels are maximal one week after ET). From previous studies, we know that the elevation in D-dimer levels occurs in a “belated” way, rather than early on, as a result of hypercoagulation and thrombosis [48]; this correlates well with the data obtained in the present study. The other hypercoagulation-sensitive assessment, the thrombodynamics, showed a maximum effect on the day of oocyte retrieval; the subsequent normalization could be related to the LMWH effect, to which the test is sensitive [29]. It is interesting that despite anticoagulation, we observed spontaneous clotting at P4 and P5 in 13% and 4% of cases, respectively. This spontaneous clotting may have several causes, such as circulating microparticles bearing factors IXa and XIa or with tissue factor [49], but at this time, we cannot reveal the main cause in our study. The absence of a correlation between the hemostasis test results during IVF treatment (P2-P4) and IVF outcome indicates the short-term effects of hormonal therapy on hemostasis. However, in the paper by Gerotziafas and colleagues [42], it was shown that a more pronounced hypercoagulant effect of hormone treatment was associated with higher chances of pregnancy. This difference with our data may be explained either by treatment protocols or population differences.

All women in the protocol received LMWH in prophylactic dosages after FP or after ET. This practice is contrary to international recommendations, but it is still implemented in several countries, including Russia. In this paper, the authors did not aim to criticize or support this practice. Notably, since heparinization in women who undergo IVF is performed with low doses of LMWH and lasts only two weeks, the frequency of complications of a hemorrhagic nature is negligible [50]. None of the patients developed any adverse effects. No laboratory assays were performed to monitor the anti-Xa level as such prophylactic doses are not recommended for monitoring. However, there are no objective data supporting this practice. According to our research, we cannot form any conclusions about the benefits or harms of prescribed treatment, since we did not have a control group that did not receive heparin.

In obstetrics and gynecology, heparin is often used not to prevent thrombosis but to improve obstetric/reproductive outcomes [51–53]. The complexity of research regarding this area is related to the fact that, in addition to the direct anticoagulant action, heparin also has anti-inflammatory and pro-invasive effects [54]. In our research, the initial hypercoagulation does not seem to be leveled by the prescribed fixed dosage of heparin in all patients, which leads to negative IVF outcomes for women with initial hypercoagulation. It is possible that this dose of LMWH is sufficient for achieving an anti-inflammatory effect but not sufficient for achieving an anticoagulant effect, which led to an impairment in the implantation of embryos in women with hypercoagulation. However, this is just a matter of speculation and currently does not have a sufficient evidence base.

One week after ET, we observed an elevated clot growth velocity in women who achieved pregnancy (Table 3). This finding is consistent with the fact that even normal pregnancy is characterized by a shift in hemostasis towards a hypercoagulation state [25]. Similar results were obtained for thrombin generation, TEG and the factor VIII level in the study by Gerotziafas et al. [42].

We followed up most of the women who became pregnant as a result of IVF. Out of 36 patients, 22 (61%) had term deliveries, and 11 (31%) had lost the pregnancy (10 women had an early loss at 4–9 gestational weeks, and 1 had an antenatal fetal death at 25 gestational weeks). Three patients were lost to follow-up.

A limitation of our study is the relatively small size of the positive IVF group (36 patients) in terms of considering the results highly significant. However, we performed our investigation on a representative sample, in which the proportion of successful pregnancies was similar to the proportion among all women of the center. The women included in the study were heterogeneous in terms of anamnesis. We did not perform an ultrasound angioscanning investigation because the analysis of the association between hypercoagulation during hormone therapy and thrombosis complications was beyond the scope of the current study. No patients showed clinical symptoms of thrombosis.

## Conclusions

Our findings are applicable to IVF with fresh ET after ICSI/IMSI. In conclusion, we report a correlation between a woman’s coagulation state and her IVF outcome. This research is potentially beneficial for the application of thrombodynamics assays for monitoring hemostasis in infertile women prior to the IVF procedure with the goal of selecting a group requiring hemostasis correction to increase the chances of pregnancy.

## Supporting information

S1 Table Blood analysis and hormone levels before IVF. (DOCX)

S2 Table Hemostasis assay parameters before IVF. (DOCX)

S3 Table ROC analysis parameters for thrombodynamics sensitivity to a negative IVF outcome. (DOCX)

S4 Table ROC analysis parameters for platelet count sensitivity to a negative IVF outcome. (DOCX)

S1 Fig Individual trends in parameter V in the thrombodynamics analysis throughout IVF treatment in women having (A) initial hypercoagulation (V>32.3), (B) initial normal coagulation. Each line represents an individual patient. The shaded area represents the 5–95% range of parameter V in the control group.
(DOCX)

S2 Fig D-dimer dynamics in patients during IVF depending on normal/high D-dimer levels before IVF. (DOCX)

S3 Fig Platelet count (A) and D-dimer levels (B) measured in groups of normal/high V values at P1. The shaded area indicates the normal ranges. NS indicates a nonsignificant difference; Mann-Whitney U test.
(DOCX)
