# Supplementary material for: An enhanced clot growth rate before in vitro fertilization decreases the probability of pregnancy
Source: PLoS One. 2019 May 23;14(5):e0216724. doi: 10.1371/journal.pone.0216724 (PMC6532853; doi:10.1371/journal.pone.0216724)
Supplement: S1 Table — (DOCX) [file pone.0216724.s001.docx]

**S1 Table.** **Blood analysis and hormone levels before IVF.**

| **Parameter** | **Reference** | **Non-Pregnant** | **Pregnant** | **P value** |
| --- | --- | --- | --- | --- |
| RBC (10^12^/L) | 3.5-4.7 | 4.5 (4.3-4.7) | 4.4 (4.2-4.6) | 0.373 |
| WBC (10^9^/L) | 4-9 | 6.2 (5.2-7.8) | 6.2 (4.6-7.3) | 0.315 |
| HGB (g/dL) | 12-15 | 13.3 (12.7-13.9) | 13.1 (12.7-13.8) | 0.795 |
| HCT (%) | 38-47 | 39.1 (37.0-41.4) | 38.9 (37.1-39.9) | 0.445 |
| PLT (10^9^/L) | 180-320 | 241 (212-290) | 203 (227-262) | **0.046** |
| Estrogen (pM) | 84-510 | 172 (156-202) | 174 (132-198) | 0.902 |
| Progesterone (nM) | 0.98-4.83 | 1.37 (0.96-1.85) | 1.65 (1.23-2.65) | 0.319 |

Continuous data: median (interquartile range). P value represents difference between non-pregnant and pregnant women (Mann-Whitney U-test). NA = not applicable.
