# Supplementary material for: An enhanced clot growth rate before in vitro fertilization decreases the probability of pregnancy
Source: PLoS One. 2019 May 23;14(5):e0216724. doi: 10.1371/journal.pone.0216724 (PMC6532853; doi:10.1371/journal.pone.0216724)
Supplement: S2 Table — (DOCX) [file pone.0216724.s002.docx]

**S2 Table.** **Hemostasis assay parameters before IVF.**

| **Parameter** | **Reference** | **Non-Pregnant** | **Pregnant** | **P value** |
| --- | --- | --- | --- | --- |
| Patients (n) | - | 89 | 36 | NA |
| *Standard assays* | | | | |
| aPTT (sec) | 25.1-36.5 | 31.5 (29.2-32.6) | 30.9 (28.3-33.1) | NS |
| Prothrombin (%) | 70-120 | 105 (97-115) | 110 (96-115) | NS |
| Fibrinogen (g/L) | 2.00-3.93 | 2.6 (2.4-3.1) | 2.6 (2.4-3.1) | NS |
| D-dimers (ng/L) | 0-250 | 109 (83-144) | 108 (85-149) | NS |
| *Thrombodynamics* | | | | |
| V (µm/min) | 23-30 | 26.3 (24.9-28.7) | 25.6 (24.2-28.5) | NS |
| D (a.u.) | 19800-26900 | 23072 (21670-24875) | 22734 (20478-25005) | NS |
| SpCl (%) |  | 6% | 0% | NA^#^ |

Continuous data: median (interquartile range); only SpCl presented as number (percentage). P value represents difference between non-pregnant and pregnant women (Mann-Whitney U-test or Fisher criterion^#^). NS = not significant, NA = not applicable.
