# Supplementary material for: An enhanced clot growth rate before in vitro fertilization decreases the probability of pregnancy
Source: PLoS One. 2019 May 23;14(5):e0216724. doi: 10.1371/journal.pone.0216724 (PMC6532853; doi:10.1371/journal.pone.0216724)
Supplement: S3 Table — (DOCX) [file pone.0216724.s003.docx]

**S3 Table. ROC analysis parameters for thrombodynamics sensitivity to a negative IVF outcome**†

| Parameter | Units | N† | AUC | AUC 95% CI | P (area=0.5) | Youden index J | Associated criterion | Sensitivity | Specificity |
| --- | --- | --- | --- | --- | --- | --- | --- | --- | --- |
| V | μm/min | 18 | 0.84 | 0.6-1.0 | 0.0042 | 0.75 | >32.3 | 75.0 | 100 |

†Only values exceeding 5-95% range for control group were used
