# Supplementary material for: An enhanced clot growth rate before in vitro fertilization decreases the probability of pregnancy
Source: PLoS One. 2019 May 23;14(5):e0216724. doi: 10.1371/journal.pone.0216724 (PMC6532853; doi:10.1371/journal.pone.0216724)
Supplement: S4 Table — (DOCX) [file pone.0216724.s004.docx]

**S4 Table. ROC analysis parameters for platelet count sensitivity to a negative IVF outcome**

| Parameter | Units | N | AUC | AUC 95% CI | P (area=0.5) | Youden index J | Associated criterion | Sensitivity | Specificity |
| --- | --- | --- | --- | --- | --- | --- | --- | --- | --- |
| PLT | 10^9^/l | 125 | 0.62 | 0.5-0.7 | 0.034 | 0.23 | >229 | 65.1 | 58.1 |
