# Supplementary material for: An enhanced clot growth rate before in vitro fertilization decreases the probability of pregnancy
Source: PLoS One. 2019 May 23;14(5):e0216724. doi: 10.1371/journal.pone.0216724 (PMC6532853; doi:10.1371/journal.pone.0216724)
Supplement: S1 Fig — Each line represents an individual patient. The shaded area represents the 5–95% range of parameter V in the control group. (DOCX) [file pone.0216724.s005.docx]

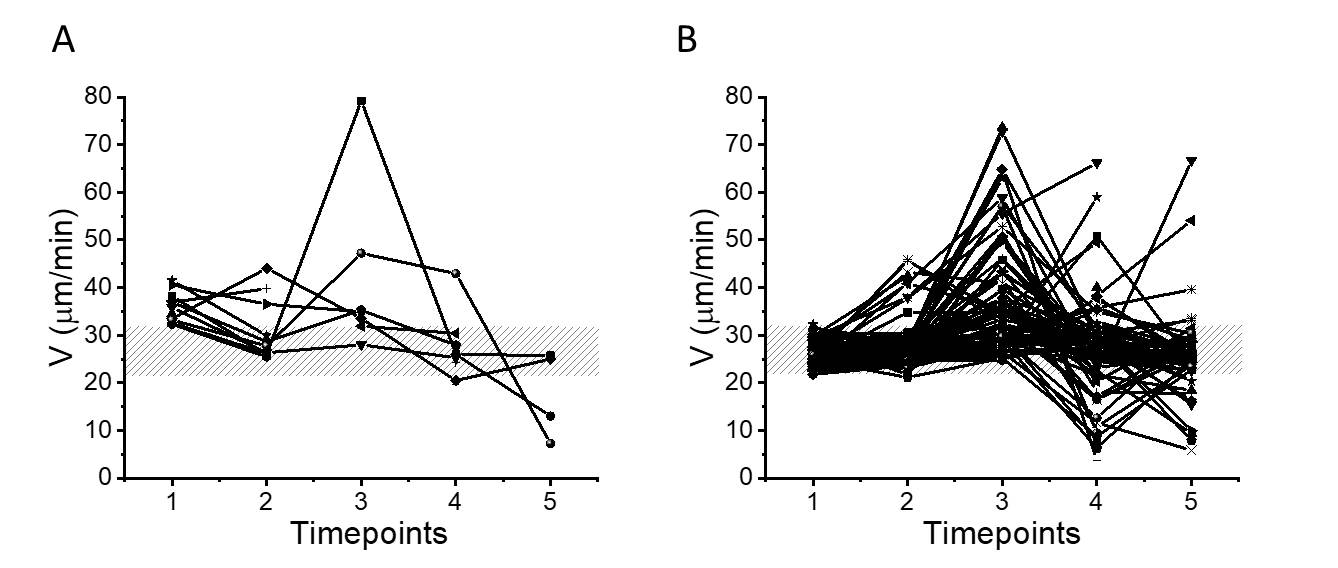


**S1 Fig. Individual trends in parameter V in the thrombodynamics analysis throughout IVF treatment in women having (A) initial hypercoagulation (V>32.3), (B) initial normal coagulation.** Each line represents an individual patient. The shaded area represents the 5-95% range of parameter V in the control group.
