# Supplementary material for: An enhanced clot growth rate before in vitro fertilization decreases the probability of pregnancy
Source: PLoS One. 2019 May 23;14(5):e0216724. doi: 10.1371/journal.pone.0216724 (PMC6532853; doi:10.1371/journal.pone.0216724)
Supplement: S2 Fig — (DOCX) [file pone.0216724.s006.docx]

**S2 Fig. D-dimer dynamics in patients during IVF depending on normal/high D-dimer levels before IVF.** Points represent: test results before IVF (P1), one week after COS starting (P2), before FP (P3), before ET (P4) and one week after ET (P5). (○) indicate plasma samples with hypercoagulation in P1; (□) indicate plasma samples with normal coagulation in P1. Mean values and SEM are presented. *, ** and *** indicate significant difference (p<0.05, p<0.01 and p<0.001, respectively; Mann-Whitney U-test) between samples with normal and high D-dimers level in P1.
