# Supplementary material for: An enhanced clot growth rate before in vitro fertilization decreases the probability of pregnancy
Source: PLoS One. 2019 May 23;14(5):e0216724. doi: 10.1371/journal.pone.0216724 (PMC6532853; doi:10.1371/journal.pone.0216724)
Supplement: S3 Fig — The shaded area indicates the normal ranges. NS indicates a nonsignificant difference; Mann-Whitney U test. (DOCX) [file pone.0216724.s007.docx]

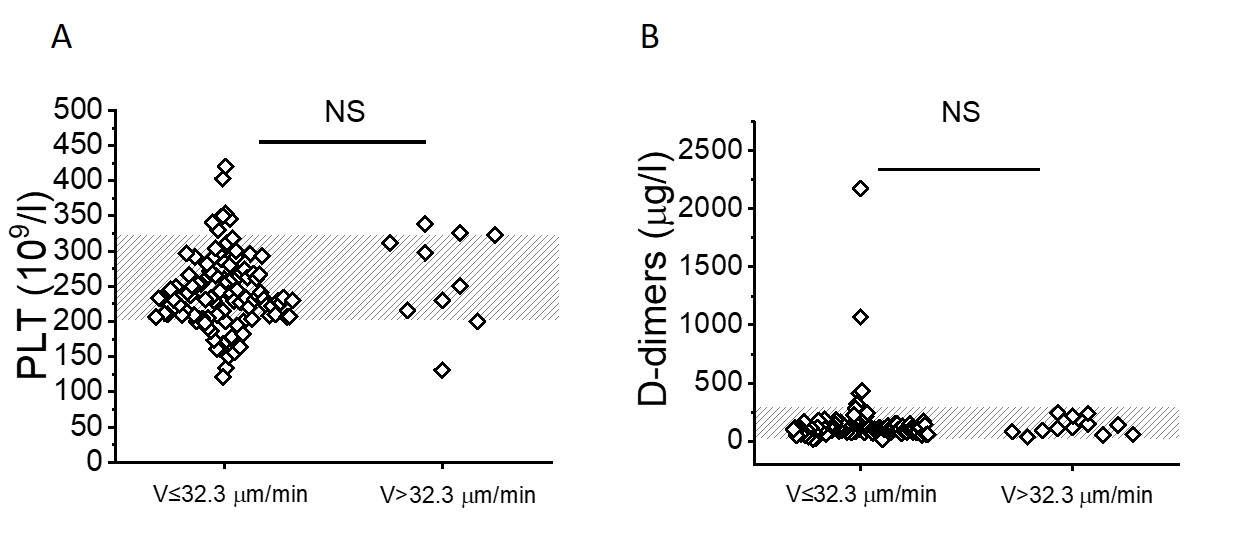


**S3 Fig. Platelet count (A) and D-dimer levels (B) measured in groups of normal/high V values at P1.** The shaded area indicates the normal ranges. NS indicates a nonsignificant difference; Mann-Whitney U test.
